# Retraction: The world’s first glyphosate-resistant case of *Avena fatua* L. and *Avena sterilis* ssp. *ludoviciana* (Durieu) Gillet & Magne and alternative herbicide options for their control

**DOI:** 10.1371/journal.pone.0321327

**Published:** 2025-03-24

**Authors:** The PLOS One Editors

Due to issues identified post-publication, PLOS re-reviewed this article [1] with support from a *PLOS One* Editorial Board member and an external reviewer. Based on the outcome of this review, the *PLOS One* Editors concluded that the article does not meet the journal’s third and fourth publication criteria. Therefore, the *PLOS One* Editors retract this article. We regret that the issues were not identified and addressed prior to the article’s publication.

The author did not agree with the retraction.
